# The Disordered Vaginal Microbiota Is a Potential Indicator for a Higher Failure of *in vitro* Fertilization

**DOI:** 10.3389/fmed.2020.00217

**Published:** 2020-06-24

**Authors:** Yao Kong, Zhaoxia Liu, Qingyao Shang, Yuan Gao, Xia Li, Cihua Zheng, Xiaorong Deng, Tingtao Chen

**Affiliations:** ^1^Department of Obstetrics and Gynecology, The Second Affiliated Hospital of Nanchang University, Nanchang, China; ^2^Department of Assisted Reproduction, Jiujiang Maternal and Child Health Care Hospital, Jiujiang, China; ^3^National Engineering Research Center for Bioengineering Drugs and the Technologies, Institute of Translational Medicine, Nanchang University, Nanchang, China; ^4^Department of Gastrointestinal Surgery, The Second Affiliated Hospital of Nanchang University, Nanchang, China

**Keywords:** high-throughput sequencing, *in vitro* fertilization, infertility, pregnancy outcome, vaginal microbiota

## Abstract

Infertility is one of the most common reproductive system diseases, and no effective method is available for its treatment. Although *in vitro* fertilization (IVF) has been widely used to enhance the clinical pregnancy outcome of infertility, the unsatisfied pregnancy rate with unknown reasons is obtained. To identify the possible cause of IVF failure, 555 patients were enrolled in the present study to determine their relevant clinical characteristics and vaginal microbiota. Our results indicated that the age and endometrium thickness significantly affected the pregnancy success rate of pregnant patients (P group) and non-pregnant patients (NP group) receiving IVF, and high values of luteinizing hormone, estrogen and progesterone were observed from P group. Furthermore, the Partial Least Squares Discriminant Analysis (PLS-DA) indicated a different microbial composition in P group and NP group, and a higher microbial abundance had been identified in non-pregnant patients compared with pregnant patients. At phylum level, a higher abundance of *Firmicutes* and *Proteobacteria*, and a lower abundance of *Actinobacteria, Fusobacteria*, and *Bacteroidetes* were obtained in pregnant patients compared with non-pregnant patients. At genus level, a lower abundance of the probiotic *Lactobacillus*, and higher abundance of pathogens *Gardnerella* and *Prevotella* were identified from non-pregnant patients. Therefore, the disordered microbiota, characterizing by the reduction of probiotics and overgrowth of pathogens in non-pregnant patients, may be used as a potential indicator for a higher IVF failure rate.

## Introduction

Infertility belongs to one of the reproductive system diseases, which is defined as having not reached at a clinical pregnancy for 12 months or more of unprotected sexual intercourse by the World Health Organization (WHO) (1). With the rapid development of human society, environmental problems (e.g., water pollution and air pollution), artificial and medical abortion, etc. greatly increased the rate of infertility (2).

Currently, Chinese traditional medicine, western medicine, combined therapy with traditional Chinese medicine and western medicine, and assisted reproductive technology are widely used to treat infertility, of which the assisted reproductive technology is the most effective treatment for infertility nowadays (3).

*In vitro* fertilization (IVF) is the most common assisted reproductive technology. Up to now, five million babies have been born through IVF technology and increases at an annual rate of 200,000 (4). However, some problems have emerged companied with the development of IVF. The outcome of IVF can be influenced by physical and physiological factors, on one hand, physical factors such as uterine contraction makes the embryo escape from the uterus (5); on the other hand, physiological factors such as age of the female patient, the age of infertility, the quality of the transplanted embryo, secretion of related hormones can also seriously affect the pregnancy rate (6, 7). In addition, studies have confirmed that during the IVF transplant operation, the pathogens of the cervix may be brought into the uterine cavity, which will also affect the IVF outcome (8). Nowadays, the rate of embryo transfer has achieved a satisfactory clinical pregnancy outcome by increasing the number of obtained eggs, the rate of fertilization and cleavage (9), while the clinical pregnancy rate is still about 30–40% (10). Therefore, it is a hot spot to increase the clinical pregnancy rate of IVF in the field of reproductive medicine.

There is growing evidence that microbes play an important role in human development, physiology, immunity, and nutrition (11) As one of the most important human-microbial habitats, the vaginal secretions and vaginal epithelial cells in vagina provide rich nutrients for the growth of a wide variety of pathogenic (e.g., *Gardnerella* and *Prevotellas*) and non-pathogenic (e.g., *Lactobacillus*) organisms (12, 13), of which *Lactobacilli* can help host to prevent vaginal infection via producing lactic acid, hydrogen peroxide, bacteriocins (14).

The human vaginal microbiota plays a vital role in maintaining the health of a women, partner or newborn (15), and studies showed that dysregulation of vaginal microbiota was connected with poor pregnancy rates, spontaneous abortion, miscarriage, and spontaneous preterm birth in human reproduction (16). Whereas, little work has been done to explore the potential connection between vaginal microbiota and IVF. Therefore, 555 female patients who underwent IVF were enrolled in the present study, and the high-throughput sequencing method was used to compare the microbial diversity of patients with successful or failed IVF outcomes.

## Materials and Methods

### Patients and Samples

Between June 2017 and December 2018, 555 consecutive infertility patients in the Jiujiang Maternal and Child Health Care Hospital of Jiangxi province were enrolled. All patients (with normal sexual life, no history of smoking, no history of bilateral ovarian surgery, the body mass index between 18 and 25 kg/m2, normal thyroid gland function, no associated contraindication to use hormone therapy) agreed to receive IVF treatment and signed informed consent for IVF. Patients coincided with the following features were excluded: interruption or discontinuation of treatment for any reason, abnormal ovarian function, abnormal cervical cytology, abnormal semen examination of partner, or other diseases combined with infectious diseases, malignant tumors, severe diabetes, severe liver, and kidney disease. The enrolled patients were mainly tubal infertility (e.g., blocked, damaged, or absent fallopian tubes, endometriosis, etc.), because it is the main cause of female infertility. However, ovarian dysfunction will cause abnormal basic hormone levels in patients, and it cannot be ruled out that the imbalance of hormone levels leads to the failure of IVF. In addition, infertility caused by male factors (such as abnormal semen, physiological diseases, etc.) is essentially not directly related to female infertility. Therefore, we excluded infertility caused by ovarian dysfunction and male factors from the enrolled criteria. In addition, in order to ensure the homogeneity of the experimental data, the IVF treatment of the infertility patients enrolled in this experiment are all used in frozen embryo transfer.

Before collecting vaginal secretion samples, it is required to ensure that no cervical treatment and no vaginal irrigation within seven days, no sexual activity within 2 days and all patients did not use any antibiotic within 15 days, so as not to affect the results of the study. Vaginal secretion samples were collected before the preparation of the endometrium, using vaginal speculum to expose the cervix, the clinician used a sterile suction tube to obtain a vaginal secretion sample from the posterior iliac crest, added 20% glycerol, and immediately frozen at −80°C until further use. Reproductive outcomes were recorded on the basis of the definition of biochemical and clinical pregnancy. According to the pregnancy outcome of IVF, the samples were divided into two groups, the pregnancy successful group (P) and the pregnancy failure group (NP). All patients were allowed to obtain medical records to obtain their reproductive history and reproductive outcomes.

## Reproductive Outcome Analysis

Fifteen days after the embryo transfer, the pregnancy result was detected by the detection of β-Human Chorionic Gonadotropin (β-HCG) in serum, and the clinical diagnosis of pregnancy was determined by detecting the pregnancy sac, embryonic bud and embryonic heartbeat using transvaginal sonography after the 6th week of pregnancy (17). Ectopic pregnancy (occurring outside the uterus), biochemical pregnancy abortion (a diagnosis diagnosed only by the detection of β-HCG in serum or urine and that does not develop into a clinical pregnancy) were excluded from the clinical pregnancy (18).

**Figure 1:**
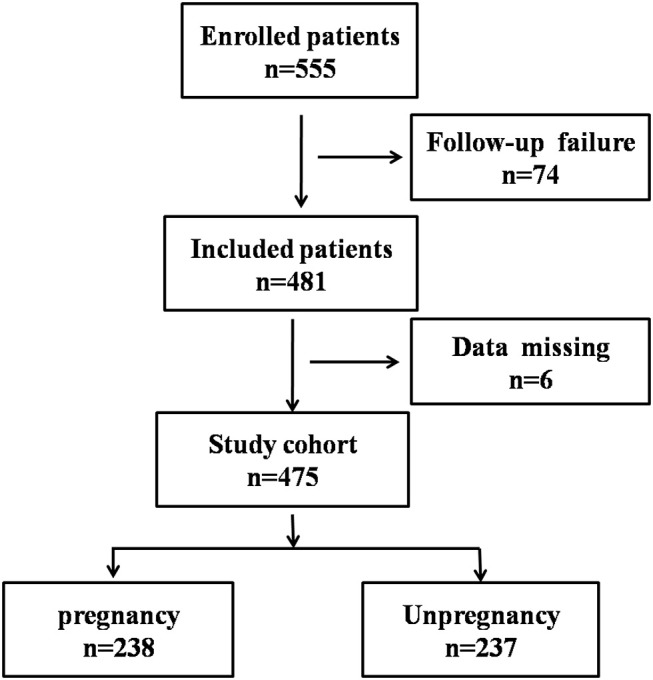
Flowchart showing the process of patient enrollment. P, pregnancy success after embryo transfer; NP, pregnancy failure after embryo transfer.

According to the pregnancy outcome of IVF, patients were divided into P group (pregnancy group) and NP group (none pregnancy group) (Figure 1), and their basic status, e.g., ages, basic hormone levels, the intimal preparation protocol, days of administration, number of embryos, and endometrium thickness, were compared in Table 1.

**Table 1:** Baseline patient demographics and characteristics.

**Variable**	**Total (*N* = 475)**	**P Group (*N* = 238)**	**NP Group (*N* = 237)**	***P*-value**
Age (years)	33.14 ± 5.57	31.89 ± 4.90	34.42 ± 5.92	0.00
**Endometrial preparation programs [(%)]**				
NC	46 (9.68)	18 (7.56)	28 (11.81)	/
COH	14 (2.95)	4 (1.68)	10 (4.22)	/
HRT	256 (53.89)	133 (55.88)	123 (51.90)	/
D+HRT	60 (12.63)	27 (11.34)	33 (13.92)	/
C+HRT	37 (7.79)	18 (7.56)	19 (8.02)	/
Unknown	62 (13.05)	38 (15.97)	24 (10.13)	/
Luteinizing hormone (mIU/ml)	8.13 (2.90–14.01)	8.93 (3.19–15.42)	7.82 (2.60–12.98)	0.52
Estrogen (pg/ml)	391.21 (216.85–1439.28)	402.84 (213.29–1501.59)	380.07 (223.82–1431.36)	0.52
Progesterone (ng/ml)	0.45 (0.30–0.67)	0.49 (0.33–0.67)	0.42 (0.29–0.67)	0.34
Taking days (days)	17 (15.00–20.00)	17.00 (15.00–20.00)	17.00 (14.50–21.00)	0.28
Endometrium thickness (mm)	9.00 (8.50–10.00)	9.20 (8.50–10.00)	8.90 (8.18–10.00)	0.02
Embryo number (*n*)	2 (1,2)	2 (2)	2 (2)	0.38

*P, pregnancy success after embryo transfer; NP, pregnancy failure after embryo transfer; p < 0.05 indicates significant difference. NC, natural cycle; COH, controlled ovarian hyperstimulation; HRT, hormone replacement therapy; D+HRT, down-regulating hormone replacement therapy; C+HRT, constant hormone replacement therapy*.

## Analysis The Vaginal Bacterial Diversity Using High-Throughput Sequencing

Thirty-seven vaginal secretions in P group and twenty-one vaginal secretions in NP group were used to compare vaginal microbial diversity using high-throughput sequencing. The steps for this technique are as follows:

DNA extraction and amplification: the genomic DNA kits (Tiangen Biotech Co., Ltd., Beijing, China) and the bead beating method were used (19) to extract DNA from samples. Then, the spectrophotometer (NanoDrop; Thermo Fisher Scientific, Inc., Waltham, MA, USA) was used to determine the concentration and quality of the purified DNA at 230 nm (A 230) and 260 nm (A 260). 520F/802R primers (520F, 5′-AYTGGGYDTAAAGNG-3′; 802R, 5′-AYTGGGYDTAAAGNG-3′) were used to amplify the V4 region of the 16S rDNA genes in each sample, and these PCR products were sequenced with an Illumina HiSeq 2000 platform (GenBank accession number PRJNA554010) sequencer in Personal Biotechnology Co., Ltd., Shanghai, China.

Sequencing and analysis: the TruSeq Nano DNA LT Library Preparation Kit (Illumina company) was used to prepare sequencing libraries and then use the Agilent High Sensitivity DNA Kit to check the quality of the library before the sequencing begins. Finally, the sequencing results were analyzed as follows: he paired-end reads were checked and removed using QIIME [Quantitative Insights Into Microbial Ecology, v1.8.0, http://qiime.org/, (20)] and USEARCH (v5.2.236, http://www.drive5.com/usearch/). Sequence analysis was subsequently performed using UCLUST software package (21), and sequences with ≥97% similarity were assigned to the same operational taxonomic units (OTU). According to the obtained OTU abundance matrix, R software was used to calculate the number of OTU Shared by each sample (group), and the proportion of OTU Shared and unique by each sample (group) was intuitively presented by Venn graph. Then, the α diversity including the observed-OTUs, the Chao1 estimator, the Shannon diversity index, the Simpson index, the ACE estimator, goods-coverage, and β diversity including PCA (Principal component analysis), PcoA (Principal coordinates analysis) and NMDS (Multidimensional scaling) were measured by Qiime software (Version 1.8.0). The weighted UniFrac distance was also measured by QIIME (Quantitative Insights Into Microbial Ecology, v1.8.0, http://qiime.org/) software package (version 1.8.0) before the cluster analysis. *P* < 0.05 showed the statistical significance. Finally, PICRUSt (Phylogenetic Investigation of Communities by Reconstruction of Unobserved States) was used to predict the metabolic function of bacteria in KEGG functional spectrum database.

### Statistical Analysis

Data handling, analyses and graphical representations were processed by Microsoft Excel, GraphPad Prism 8 (https://www.graphpad.com/) and SPSS 23.0 software (SPSS Inc., Chicago, IL, USA). Statistical significance was determined using a Student's test, one-way or two-way ANOVA and were annotated using the international convention related to the statistical representation. For continuous variables that satisfy a normal distribution, such as age, the data will be reported as mean ± SD. Otherwise, it is expressed in quartiles.

## Results

### Patient Enrollment and Baseline Characteristics

The patient enrollment process is described in Figure 1. In total, 555 patients receiving IVF were enrolled between June 2017 and December 2018, 74 patients were excluded due to follow-up failure. Finally, 481 patients were included in the study and 6 patients were excluded for data missing. In the end, 238 patients were divided into pregnancy group (P) and 237 patients were divided into none pregnancy group (NP). According to the patient's physical condition, different endometrial preparation programs were selected, including NC (natural cycle), COH (controlled ovarian hyperstimulation), HRT (hormone replacement therapy), D + HRT (Down-regulating hormone replacement therapy), C + HRT (Constant hormone replacement therapy).

As shown in Table 1, the age (*p* = 0.00) and endometrium thickness (*p* = 0.02) had a strong connection with the pregnancy outcomes. Although no significant difference were observed between IVF successes with luteinizing hormone (*p* = 0.52), estrogen (*p* = 0.52), progesterone (*p* = 0.34), taking days (*p* = 0.28) and embryo number (*p* = 0.38), high values of luteinizing hormone (8.93 vs. 7.82), estrogen (402.84 vs. 380.07), and progesterone (0.49 vs. 0.42) were obtained from P group.

### Alpha-Diversity and Comparative Analysis of the Vaginal Microbial Community Between P and NP Groups

To further explore the potential factors on IVF successes, vaginal sections of 58 patients were randomly sampled from the enrolled 475 infertile patients, and these samples were divided into P group (*N* = 37) and NP group (*N* = 21) based on their pregnancy situations.

**Figure 2:**
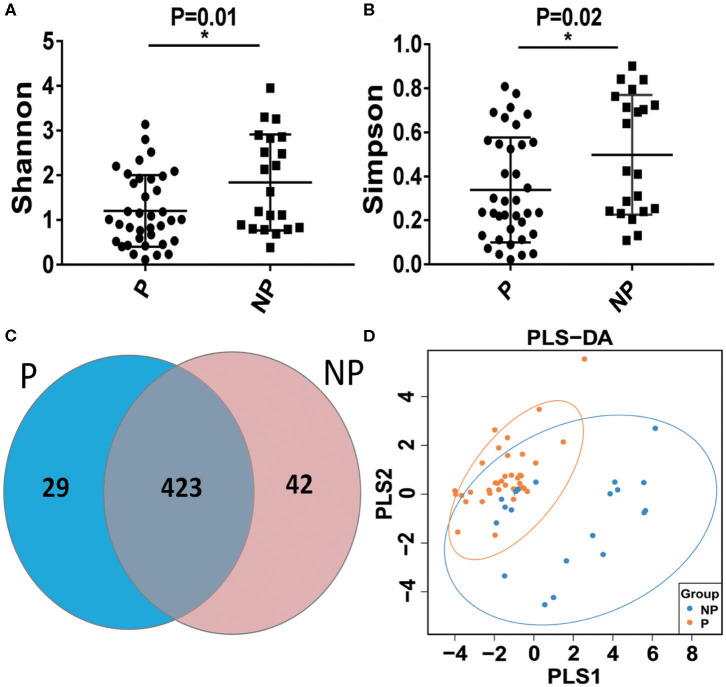
Alpha-diversity and comparative analysis of the vaginal microbial community in the P and NP groups. The Shannon index **(A)** the Simpson index **(B)** the Scalar-Venn **(C)** the PLS-DA **(D)**. P, pregnancy success after embryo transfer; NP, pregnancy failure after embryo transfer; *means *P* < 0.05, *p* < 0.05 indicates significant difference.

The vaginal microbial diversity in P group and NP group were compared using high-throughput sequencing. Our results indicated that a total of 2,624,974 filtered clean reads (4,525.81 reads/sample) and 3,413 OTUs were obtained from all the samples (data not shown). As shown in Figures 2A,B, a higher Shannon index and Simpson index was obtained in NP group compared with P group. In Figure 2C, the Scalar-Venn results indicated that there were 452 OTUs and 465 OTUs in P group and NP group, and 423 common OTUs were observed, which occupied 93.58% (423/452) and 90.97% (423/465) in P group and NP group, respectively. In addition, the Partial Least Squares Discriminant Analysis (PLS-DA) result indicated that most dots in P groups scatted far away from that in NP groups (Figure 2D).

### Comparison of the Vaginal Microbial Community Between P and NP Groups at Phylum Level and at Genus Level

**Figure 3:**
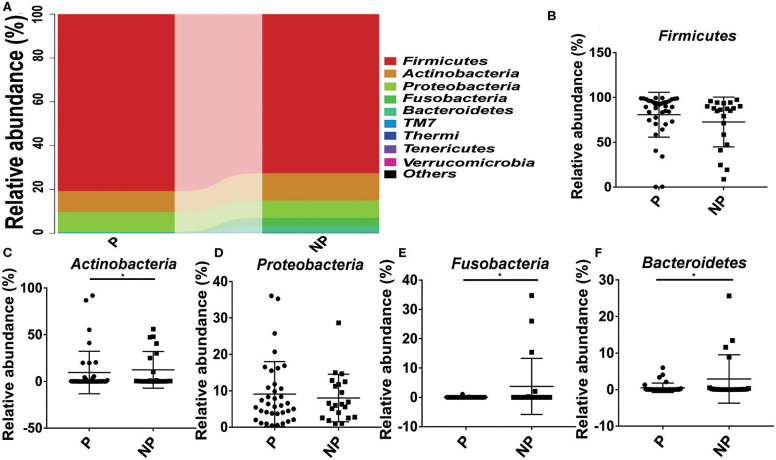
Comparison of the microbial community of vaginal between P and NP groups at phylum level. **(A)** Cumulative bar charts of the main taxa at phylum levels in P and NP samples. *Firmicutes* **(B)**, *Actinobacteria* **(C)**, *Proteobacteria* **(D)**, *Fusobacteria* **(E)**, *Bacteroidetes* **(F)**. P, pregnancy success after embryo transfer; NP, pregnancy failure after embryo transfer; *means *P* < 0.05, *p* < 0.05 indicates significant difference.

The top 10 microorganism populations at the phylum level were shown in Figure 3A, and *Firmicutes, Actinobacteria, Proteobacteria, Fusobacteria*, and *Bacteroidetes* constituted the most common dominant phyla in P group (80.77, 9.52, 9.18, 0.04, and 0.40%, respectively) and NP group (72.61, 12.41, 8.01, 3.75%, respectively), which accounted for 99.91 and 99.83% of the total sequencing number in these two groups. Compared with the NP group, a higher abundance of *Firmicutes* (80.77 vs. 72.61%) and *Proteobacteria* (9.18 vs. 8.01%), and a lower abundance of *Actinobacteria* (9.52 vs. 12.41%, *p* = 0.03), *Fusobacteria* (0.04 vs. 8.01%, *p* = 0.02), and *Bacteroidetes* (0.40 vs. 3.75%, *p* = 0.03) were observed in P group (Figure 3).

**Figure 4:**
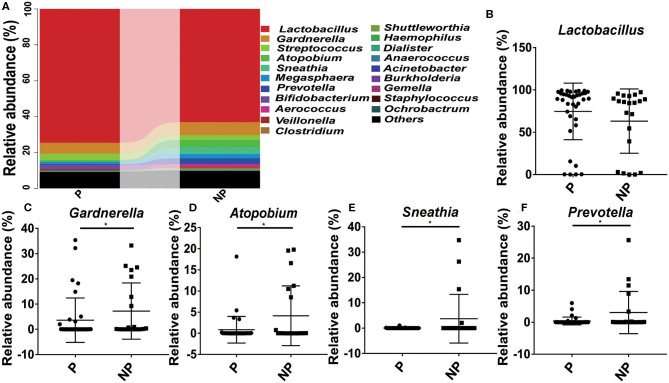
Comparison of the microbial community of vaginal between P and NP groups at genus level. **(A)** Cumulative bar charts of the main taxa at genus levels in P and NP samples. *Lactobacillus* **(B)**, *Gardnerella* **(C)**, *Atopbium* **(D)**, *Sneathia* **(E)**, *Prevotella* **(F)**. P, pregnancy success after embryo transfer; NP, pregnancy failure after embryo transfer; *means *P* < 0.05, *p* < 0.05 indicates significant difference.

At genus level, it seems that the pregnancy have a strong connection with the higher abundance of probiotics *Lactobacillus* (74.61 vs. 63.09%), and the lower abundance of pathogens *Gardnerella* (6.03 vs. 7.24%, *p* = 0.03), *Atopobium* (0.84 vs. 4.14%, *p* = 0.02), *Sneathia* (0.03 vs. 3.75%, *p* = 0.02), and *Prevotella* (0.38 vs. 3.02%, *p* = 0.04) (Figure 4).

### Biological Processes and Molecular Function of the P and NP Groups

**Figure 5:**
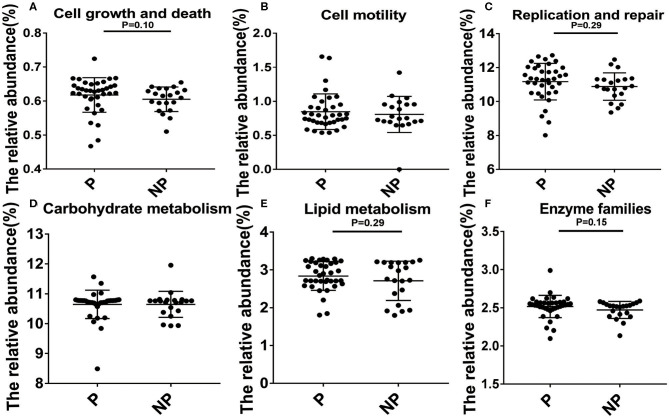
Biological processes and molecular function of the P and NP groups. Analysis of differences between P and NP groups in cell growth and death **(A)**, cell motility **(B)**, replication and repair **(C)**, carbohydrate metabolism **(D)**, lipid metabolism **(E)**, enzyme families **(F)**. There was a significant difference in the function of the microbiota between the P and NP groups (*P* < 0.05). P, pregnancy success after embryo transfer; NP, pregnancy failure after embryo transfer.

In the end, Kyoto Encyclopedia of Genes and Genomes (KEGG, the major public pathway-related database) is used to compare the effect of altered microbial changes on host functions. As shown in Figure 5, it seems that the vaginal microbiota in P group had a closer connection with cell growth and death (0.63 vs. 0.62%, *P* = 0.10), replication and repair (11.33 vs. 10.90%, *P* = 0.29), lipid metabolism (2.85 vs. 2.76%, *P* = 0.29), and enzyme families (2.54 vs. 2.52%, *P* = 0.15) compared with NP group, and the microbial difference in these two group had little effect on cell motility and carbohydrate metabolism (Figure 5).

## Discussion

The human uterus cavity is traditionally considered to be sterile, while the vaginal microbiota plays an important role in fending against pathogens (22). Emerging evidence demonstrates the presence of bacteria in the vagina is generally accepted (22, 23), but the presence of bacteria in the uterine cavity is still controversial due to limitations in identification methods. It is well known that the vaginal microbiota is the most important component of the vaginal environment (24), and the correlation between vaginal microbiota and infertility, premature birth, etc. has been gradually valued (25, 26).

In the present study, 555 patients who received IVF were enrolled and our results indicated that the age and endometrium thickness significantly affected the outcome of the IVF, and higher values of luteinizing hormone, estrogen and progesterone were obtained from P groups although these hormones had been adjusted to normal levels before the IVF for patients with abnormal hormones. Similarly, studies conducted by Grondahl ML et al. confirmed that woman's age did pose significant influence on early embryo morphological development (27). To better explore the potential reasons for IVF failure, high-throughput sequencing method was firstly and directly used to explore the microbial diversity among different outcomes of IVF, which indicated that the overgrowth of non-*lactobacillus* bacteria in the vagina, such as *Gardnerella, Atopbium, Prevotella* and so on, was related to the negative effects of reproductive function.

Infertility has become a social issue. Considering the ineffective of traditional medicines and surgery, IVF technology brings hope to infertile patients. However, the low success rate and high abortion rate for IVF bring tremendous pressure and pain to countless families. Although related factors including age, endocrine, immune, and infection have been recognized (28), little work is done to explore the connection between vaginal microbiota and IVF.

As we know, the vaginal microbiota is one of the simplest symbiotic bacterial communities in the human body, and plays an important role in maintaining micro-ecological balance, exerting space-occupying protection and colonization resistance (29). Normally, pathogens and probiotics in a woman's vagina are usually in a state of homeostasis. When the balance is disrupted, local inflammation, and pathogens will spread upward into the uterine cavity, increasing the risk of uterine infection (30). *Lactobacillus* is the most common probiotics in the vagina, the lactic acid and fatty acids produced by *Lactobacillus* acidify the environment to pH ≦ 4.5 in the vagina and inhibit the growth of other pathogenic or dysbiotic bacteria in the vagina, maintaining a healthy vaginal environment (31). Studies have shown that the vaginal microbiota changes from being *Lactobacillus* spp. dominated to a more heterogeneous and complex environment with anaerobic bacteria, such as *Gardnerella* spp. and *Prevotella* spp., was closely associated with leucorrhea abnormalities, pelvic inflammatory diseases, spontaneous abortions and premature deliveries (32, 33).

Previous studies have found that chlamydia trachomatis detected in the vagina of women receiving IVF correlates with adverse pregnancy outcome (34). The main reason is to take into account that IVF technology involves transfer of embryos through the vagina into the uterus by a catheter, which may bring the vaginal microbiota (including pathogens) into the uterus during the embryo implantation process (35), the direct production of microbial metabolites can produce related compounds which are capable of inducing key cellular pathways in endometrial epithelial cells, which will affect implantation rates and pregnancy outcomes (36). In fact, increasing evidence indicates that bacterial contamination of the uterine cavity with transvaginal embryo transfer can negatively affect the implantation rates and the pregnancy outcome (37). In the present study, high-throughput sequencing method was applied to compare the microbial composition between P group and NP group. The result of the Shannon index and Simpson index showed a lower bacterial abundance in P group compared with NP group. This may be due to the replacement of dominance of *Lactobacillus* with aerobic bacteria (e.g., *Gardnerella* vaginalis, *Prevotella* vaginalis, *Atopobium* vaginalis) (38, 39). It has been reported that the neuraminidase produced by the *Gardnerella spp*. and *Prevotella spp*. can destroy the envelope of cervical epithelial cells, and the barrier between the cervix and the vagina is weakened or disappeared, which greatly increases the risk of ascending infection of pathogenic bacteria (40). Therefore, the significant reduction of *Lactobacillus* and the overgrowth of *Gardnerella* and *Prevotella* disrupts the microbial homeostasis, which destroys the situation of embryo implantation (the key period of reproduction).

Currently, the IVF treatment for infertility is quite expensive for most of patients, and once the treatment is failed, it will cause physical and mental harm to patients. So, there is an urgent need to predict biomarkers for pregnancy outcomes in women receiving IVF. In our study, we found that the reduction of *Lactobacillus* and the overgrowth of *Gardnerella, Atopbium* and *Prevotella* possessed strong connection with the success of IVF. Interestingly, these correlations were even more significant following the overgrowth of the *Gardnerella, Atopbium* and *Prevotella* genus, as patients with high incidence of *Gardnerella, Atopbium* and *Prevotella* either failed to be pregnant after embryo transfer or experienced abortion. Therefore, it seems worthwhile to discuss the role of vaginal microbiome in IVF, and prove their potential as biomarker to predict the outcomes of IVF.

However, some limitations should be taken note. One limitation of this study is the various time points for vaginal secretion collection and the no comparison of the vaginal microbiota among different ages. In previous studies, the female age has been considered to be one of the main limiting factors for fertility and reproductive outcomes (41), and the advanced maternal age may interfere with the process of aging of oocytes, which leads to the abnormal fertilization of the egg and the abnormal normal development of the blastocyst, such as polyspermy, division arrest, implantation failure and miscarriage (27). Although studies confirmed that the various vaginal microbiota between healthy women and women of premenopausal and postmenopausal (42), little work is done to explore their effect on pregnancy, therefore we cannot exclude if the different vaginal microbiota in women of different ages also play a key role for the failure of IVF. In the present study, samples were collected at different time points due to the uncontrolled time for fertility treatment, this may have an impact on the vaginal microbiota due to hormone therapy or periodic fluctuations (43). In addition, the hormone levels for patients receiving IVF have been artificially adjusted before or/and during the IVF treatment, which make the hormone levels be a irrelevant factors for the success of IVF, although hormone plays an important role embryo implantation and development (44).

## Conclusion

In the present study, high-throughput sequencing method was firstly used to explore the microbial diversity among different outcomes of IVF female. Our group found that the age, endometrial thickness, reduction of *Lactobacillus*, and the overgrowth of *Gardnerella, Atopbium*, and *Prevotella* possessed strong connection with the success of IVF, and the bacterial intervention via restoring vaginal microbial diversity to “health” status may be a good choice to enhance positive reproductive outcomes of IVF.

## Data Availability Statement

The datasets generated for this study can be found in the GenBank, accession number PRJNA554010.

## Ethics Statement

The studies involving human participants were reviewed and approved by The Second Affiliated Hospital of Nanchang University and Jiujiang Maternal and Child Health Care Hospital and this project was registered at Chinese Clinical Trial Registry (NO. ChiCTR1900022537). The patients/participants provided their written informed consent to participate in this study.

## Author Contributions

TC, ZL, and XD designed the study. YK, XL, QS, YG, and CZ carried out the experiments. TC, ZL, YK, QS, YG, CZ, and XD analyzed the references and wrote the manuscript. All authors contributed to the article and approved the submitted version.

## Conflict of Interest

The authors declare that the research was conducted in the absence of any commercial or financial relationships that could be construed as a potential conflict of interest.
